# Notes on Preparations for the Sick

**Published:** 1896-03

**Authors:** 


### Notes on Preparations for the Sick.

**Ingluvin; Bromo-soda; Aloin Parvules; Lithia Tablets; Pil. Peristaltic; Pil. Cascara Cathartic; Pil. Chalybeate Comp.**—Wm. R. Warner & Co., Philadelphia.—Samples of these have been sent to us by Messrs. F. Newbery & Son. They are all excellent preparations, and are for the most part too well known to need description. The **Parvules** are supplied in cases with forty varieties; they are quickly dissolved; and the dose is small, in order that it may be given at short intervals. The **Lithia Tablets** are for making an effervescing dose of fresh lithia water. The **Pills** are perfect.

**Thyroid Powder Palatinoids.**—Oppenheimer, Son & Co., Ltd., London.—The palatinoid appears to especially adapt itself to the administration of an animal extract. The **Thyroid Powder**, being hermetically sealed in a jujube covering, is not so prone to decomposition as when supplied in dry powder or pellet. Each palatinoid contains five grains of the fresh gland.

**Tabloids: Alkaline and Antiseptic** (Dr. Carl Seiler's formula).—Burroughs, Wellcome & Co., London.—These tabloids may be dissolved in water, and a solution is at once ready for nasal irrigation or for gargling the throat. They are composed of sodium bicarbonate, benzoate, biborate and salicylate, with thymol, menthol, and ol. Gaultheriæ. We have not been in the habit of using such an elaborate combination as this; but it is obvious that the tablolds should be of much service in cases where an alkaline antiseptic is indicated.

---

**Liq. Quininæ Salicylatis; Pastils.**—Cooper & Co., London.—Each fluid drachm of the **Liquor** contains one grain of natural salicylate of quinine. It forms a clear and elegant solution, which when diluted with water becomes opalescent and tastes much like orange bitters. Its uses are obvious.

Messrs. Cooper & Co. have sent us several preparations made with Iceland Moss Jujube as a basis, and to which they have given the name of **Pastils.** They are firm and palatable, and contain such drugs as phenacetin (gr. v.), sulphonal (gr. v.), bismuth (gr. iii.), red gum (gr. j.), and the usual components of throat and cough lozenges.

---

**Mist. Damianæ Co.**—C. J. Hewlett & Son, London.—This mixture is worthy of the appellation "an elegant preparation." It contains fluid extract of damiana in conjunction with nux vomica and bitter aromatics, and is especially intended for cases of nervous exhaustion, chronic alcoholism, &c. This kind of drug is open to great abuse, but nevertheless it has a legitimate range of utility, and will often be of some service in cases of functional nerve diseases, as also in the early stages of organic diseases of the brain and spinal cord.

---

**Terrōl.**—The Terrōl Co., London.—This is described as a tastleless substitute for cod liver oil, to which it is said to be superior, being tasteless and inodorous, digestible and non-nauseating. "It has also the advantage of antiseptic properties, rendering it of great value for the cure and prevention of many diseases of bacterial origin." We question if these statements will survive criticism. It may be a substitute for cod liver oil from the mere mechanical point of view of lubrication, but the difficulty of absorption of petroleum must render it of no value as a food. It may give mechanical relief in cases of bronchial catarrh, and it may possibly assist the processes of gastric and intestinal digestion, but its negative properties of taste and smell will not compensate for the absence of all the other properties of cod liver oil.

---

**Grape Jelly.**—The Viking Food and Essence Co., London.—This is prepared solely with pure grape juice evaporated *in vacuo*, and is specially recommended as "a substitute for grapes to those who are unable to undergo the grape-cure treatment abroad." We wonder, on reading this, whether the inspection of a few photographs of Alpine snow-peaks may have the same invigorating effect as climbing them. The grape cure is doubtless useful, especially when the patients eat eight or ten pounds a day in place of an ordinary diet; but surely the grapes are only one adjunct to the programme of the grape cure. Is not this jelly a feeble caricature of treatment? As an ordinary dietetic jelly it is good, both in appearance and flavour; but we must decline to place it in our *armamentarium* for the treatment of disease.

---

**Peptenzyme.**—Reed & Carnrick, New York.—This new digestive presents in "functional physiological activity all the digestive agents of the animal economy: stomach, pancreas, spleen, salivary and Brunner's glands, and Lieberkuhn's follicles, and—free nuclein—the tissue builder of the organism."

These elegant tabloids, containing five grains each of this omnipotent powder, ought to abolish dyspepsia from our bodies and dyspeptics from our consulting rooms. We are not quite clear as to the correctness of the physiology which underlies its composition, or as to the magical virtues which are just now being associated with the name Nuclein.

---

**Coca Wine Essence.**—Buxton & Co., Clifton.—This special preparation of Coca leaves is miscible in any proportion with wine, milk, or water; and one drachm represents the full medicinal value of one drachm of the finest green Nuxillo Coca leaves. We cannot describe the fluid as pleasant to the palate; but it has a strong odour and taste of the well-known coca leaf, of the properties of which it must partake.

---

**Bovril for Invalids; Bovril Beef Jelly; Bovril Chocolate; Small Bovril Tablets.**—Bovril Limited, London.—Some of these excellent preparations of the company presided over by Lord Playfair are thoroughly well known and appreciated; others deserve to be better known than they are. "Albumen and fibrine are the *desiderata* in these preparations. They cannot properly be restored to meat extracts or beef tea except by the process of comminuting the lean meat until it resembles very fine flour, and adding this to a special blend of meat extract. Without albumen and fibrine, beef tea is a mere stimulant; with albumen and fibrine, the essentials are provided, and this combination of the stimulative with the nutritive constituents of beef—of extract of meat with albumen and fibrine—is perfectly accomplished in Invalid Bovril, giving a perfect food in a form easy of digestion and assimilation. We may say that the difference between Invalid Bovril and ordinary Bovril lies in the former being more concentrated, and containing less seasoning; apart from that, the method of preparation is exactly the same."

We have found that a solution of the **Invalid Bovril** in warm water gives an abundant precipitate of albumen on boiling, and also with picric acid: a solution of average strength, as ordinarily taken for food, showed the presence of one gramme per litre of albumen as estimated in the picric acid tube. Microscopically, the sediment was found to consist of granular *débris* of muscular tissue, many of the fragments showing the well-marked striations of healthy muscular fibres. The **Jelly**, mounted in glass bottles, has an excellent appearance and flavour. It is extracted by gentle heat, has no added water, and is not contaminated by contact with metals. It is a very useful and convenient preparation to keep by the bedside at night, and when stimulating concentrated nourishment is needful. It makes excellent beef tea, and will be relished by the most prejudiced critic. The **Bovril Chocolate** differs little in flavour from the ordinary kind; but the tablets must contain more actual nourishment in proportion to the amount of Bovril they contain—of this we could find no mention. The **Small Tablets** are very convenient. Each contains sufficient Bovril to make a teacupful of the ordinary beef tea. A box of these carried in the pocket might be very useful in emergencies.

---

**Marza Wine.**—The Marza Company, Ltd., London.—This combination of port wine with iron, phosphorus, coca, and pepsine, must have a somewhat complicated chemical composition, which will scarcely be a guide to its therapeutic activity. It is an agreeable beverage, and as a medicine is infinitely more pleasant than most medicinal fluids. Its specific gravity is over 1030, it mixes readily with water, and does not precipitate with picric acid. Its ingredients are a sufficient indication of what its dietetic and medicinal virtues are likely to be.

---

**Soda Water; Potash Water; Lithia Water; Brighton Seltzer Water; Lemonade; Ginger Ale; Orange Champagne.**—Idris and Co., Ltd., London.—We have much pleasure in calling attention to these productions, samples of which we have received. They are of excellent quality. Each pint of the **Soda Water** contains four grains of the bicarbonate. The **Potash Water** is of the British Pharmacopœia strength. The **Lithia Water** has five grains to the half-pint. The **Orange Champagne** is a particularly pleasant drink.

---

**Purest Chloroform.**—Salamon & Co., Ltd., Rainham.—This is an excellent specimen of chloroform, which we have used as an anæsthetic and found to be quite unirritating.

---

**Compressed Ophthalmic Discs.**—John Wyeth and Brother, London.—In these we have a well-selected series of small tabloids and gelatine discs, which contain in proper dosage the various medicaments generally used in ophthalmic practice.

**Tannopumilene; Rose Bloom; Tongue Cloths.**—Arthur and Co., London.—**Tannopumilene** is intended for unhealthy or excessive perspiration, and is useful for perspiration of the feet. **Rose Bloom** is a mixture of soluble reds made to match the tint of the skin, and is intended, we presume, for painting ladies' faces: it is said to be quite harmless; but, although we are assured by the makers that samples of it were accepted with avidity by members of the medical profession at this year's museum in London, yet we are not disposed to recommend it. **Tongue Cloths** have been prepared at the suggestion of a laryngologist, and will serve a useful purpose.

